# An Innovation-Driven Approach to Specific Language Impairment Diagnosis

**DOI:** 10.21315/mjms2021.28.2.15

**Published:** 2021-04-21

**Authors:** Yan Huan Ch’ng, Mohd Azam Osman, Hui Ying Jong

**Affiliations:** 1 School of Computer Sciences, Universiti Sains Malaysia, Pulau Pinang, Malaysia; 2 School of Humanities, Universiti Sains Malaysia, Pulau Pinang, Malaysia

**Keywords:** specific language impairment, automation, diagnosis

## Abstract

**Background:**

Specific language impairment (SLI) diagnosis is inconvenient due to manual procedures and hardware cost. Computer-aided SLI diagnosis has been proposed to counter these inconveniences. This study focuses on evaluating the feasibility of computer systems used to diagnose SLI.

**Methods:**

The accuracy of Webgazer.js for software-based gaze tracking is tested under different lighting conditions. Predefined time delays of a prototype diagnosis task automation script are contrasted against with manual delays based on human time estimation to understand how automation influences diagnosis accuracy. SLI diagnosis binary classifier was built and tested based on randomised parameters. The obtained results were cross-compared to Singlims_ES.exe for equality.

**Results:**

Webgazer.js achieved an average accuracy of 88.755% under global lighting conditions, 61.379% under low lighting conditions and 52.7% under face-focused lighting conditions. The diagnosis task automation script found to execute with actual time delays with a deviation percentage no more than 0.04%, while manually executing time delays based on human time estimation resulted in a deviation percentage of not more than 3.37%. One-tailed test probability value produced by both the newly built classifier and Singlims_ES were observed to be similar up to three decimal places.

**Conclusion:**

The results obtained should serve as a foundation for further evaluation of computer tools to help speech language pathologists diagnose SLI.

## Introduction

Specific language impairment (SLI), also known as developmental language disorder (DLD) is a disorder which causes delayed language development without physical nor intellectual inhibiting factors. Individuals suffering from SLI experience difficulties producing words verbally, learning new words and making conversation. More prominent symptoms also include long delays in order to comprehend a written or spoken sentence (1). Being especially common among children and adolescents, SLI affects approximately 7% to 8% of children in kindergarten worldwide, and the problems introduced by SLI can persist into adulthood if it is not diagnosed and treated correctly (1). Traditionally, SLI diagnosis is carried out manually by speech-language pathologists and therapists. Standardised tests involving questionnaires began to be used in schools to screen for cases of language impairments. Following the integration of technology in the medical field, sophisticated tools such as eye trackers and electroencephalogram (EEG) biosensors became more accessible, which allowed them to more accurately diagnosis SLI. However, SLI diagnosis remains a daunting task because eye trackers, EEG biosensors and the like are expensive and not readily available to all therapists and researchers. A brief review reveals that mid-range eye trackers can cost up to USD10,000 and high-end eye trackers mainly used for research purposes typically cost even more (2). Even low-end eye trackers which are not recommended for research use cost as much as USD1,000 (2). EEG biosensors which evaluate electrical activity in the brain cost up to USD25,000 and are mainly available only in professional health facilities such as hospitals. Other problems include the lack of integration among tools used to diagnose SLI, as well as the lack of automation or computer-aided systems to help increase the effectiveness and efficiency of SLI diagnosis. In order for a complete and thorough diagnosis to take place, the pathologist or therapist in charge has to learn how to utilise different tools, many of which are not within their field of expertise. This often makes the already complex task of diagnosing and treating SLI unnecessarily difficult and even chaotic. As a result, the current process of diagnosing with SLI is inefficient. Although major effort has been placed in building an automated screening tool for SLI-related disorders in the past decade, not much has been put to practical use locally because speech in children differs throughout their development and the expert knowledge of speech-language pathologists required to apply appropriate concepts on a per-case basis cannot be readily duplicated by machines. As a result, SLI diagnosis still requires time-intensive assessments, which are often not administered until parents, doctors or teachers notice the abnormalities in children suffering from SLI (3).

Based on the problems and inconveniences which have been previously mentioned, it is clear that solving the issue requires, firstly, the dependency on costly equipment which is not widely available in our everyday lives to be eliminated from the SLI diagnosis process. Secondly, a computerised system integrated with all the tools required to diagnose SLI should be developed to at least semi-automate the SLI diagnosis process. The present study proposes the use of an innovation-driven approach to enhance and semi-automate existing SLI diagnosis procedures. To end this, two hypotheses were raised, corresponding to the two mentioned goals, respectively.

H1: The use of costly equipment in the SLI diagnosis process can either be replaced or eliminated.
  - Eye-tracking hardware which is used to infer the gaze location of patients can be replaced via software or computer vision algorithms.
H2: An integrated, computerised system or tool to assist speech-language pathologists in SLI diagnosis can be built.
  - Manual SLI diagnosis procedures, such as the binary picture matching task, can be automated via computer programmes to reduce diagnosis cost and increase diagnosis accuracy.
  - Calculations and statistical methods for research and practice in neuropsychology can be applied and integrated into the computer programme.

With respect to H1-a, H2-a and H2-b, several experiments with different evaluation strategies have been carried out in an attempt to affirm the validity of the hypotheses which has been put forth. The rest of this paper focuses on explaining these evaluation strategies, the results of the experiments and discussions pertaining to the results.

## Methods

### Software Tools

Gaze behaviours have been studied using eye tracking with those suffering from SLI (4). Eye tracking is a common method for understanding human attention in psychology experiments, human-computer interaction studies and medical research (5). Prior research on state-of-the-art algorithms to achieve similar goals has revealed that eye tracking can be achieved without the use of hardware specific to eye tracking. One of the tools for eye tracking without hardware is WebGazer.js.

WebGazer.js is a self-calibrating eye-tracking JavaScript library that uses typical lowcost webcams found on personal computers to infer the eye-gaze locations of web visitors on a page in real time. The way web visitors interact with the web page using a point-and-click device is mapped to the features of the eye and positions on the screen via regularised linear regression (5). Another library which can be integrated with WebGazer.js to mimic the functionality of an eye tracker is heatmap.js.

The heatmap.js library is self-explanatory. Based on input coordinates, heatmap.js is capable of creating a canvas and drawing heat maps which are virtually the same as eye tracker outputs (6). The innovative combined usage of WebGazer.js and heatmap.js is anticipated to a sufficient replacement for eye trackers and all that is required would be a standard computer webcam. Apart from the mentioned libraries, pupil detection and gaze tracking algorithms in themselves are also popular topics of study and can be implemented using other libraries such as, OpenCV or even MATLAB.

## Evaluation Methodologies

### WebGazer Accuracy Test

**Figure 3:**
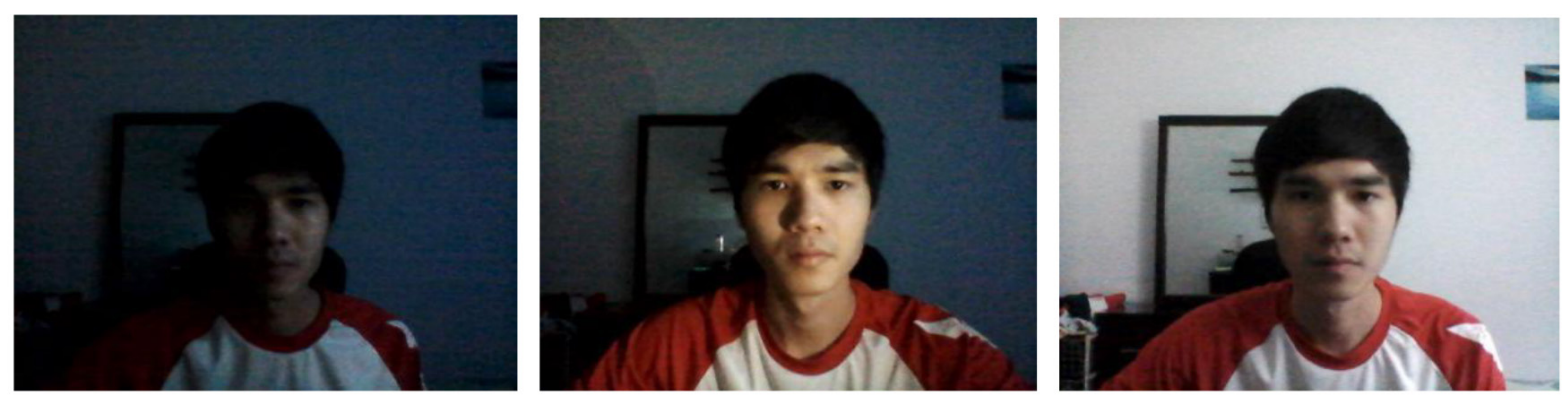
Lighting conditions (left to right: Dark, face-focused lighting, global lighting)

An original study which delineates the development and evaluation process of WebGazer claims up to 100px in terms of accuracy given sufficient lighting (5). In the following accuracy test which has been conducted, this evaluation is refined by testing WebGazer under three specific lighting conditions separately to identify the optimal lighting setting under which WebGazer, when applied for gaze tracking purposes in a computerised SLI diagnosis setting, would perform best. Figure 3 below shows the three different lighting conditions under which the WebGazer gaze tracking component was tested, via a USB Video Class Video Graphic Array (UVC VGA) WebCam at 640×480 resolution.

The three different lighting conditions are shown in Figure 3: dark, face-focused lighting and global lighting. For face-focused lighting, white light was emitted from a light source placed in front of the webcam in a dark room. In terms of accuracy measurement, accuracy was primarily derived from the distance function, wherein the distance between the predicted gaze location and the actual gaze location is calculated with respect to the on-screen coordinates. The Pythagoras theorem was used to calculate the distance between the two points given the x,y coordinates of both points.

$$\text{Distance},\text{D}(\text{f})=\surd ({\left(\text{x}1-\text{x}2\right)}^{2}+{\left(\text{y}1-\text{y}2\right)}^{2})$$

As such, the average distance of each gaze tracking session is calculated via this equation.

$$\bar{u}=\sum_{i=l}^{n}\frac{D(i)}{n}$$

given *n* = total coordinate points predicted by WebGazer in a single gaze tracking session. In this accuracy test, *n* is selected to be a constant of 100 — that is, 100 gaze coordinates are predicted in each test session. Given the mean distance, the accuracy is derived by the following equation:

$$acc=\frac{1000-\bar{u}}{1000}$$

which operates under the assumption that any prediction beyond the distance of 1000px is of 0 accuracy. The smaller the mean distance between the predicted point and the actual point, the closer the accuracy is to 1. Given the above equation, the documentation of WebGazer claims 0.9 accuracy under optimal conditions.

### Automated Versus Manual Duration Judgement Test

By observing the existing SLI diagnosis procedures and the steps involved in order to arrive at the diagnosis results, it is clear that there are a multitude of ways to diagnose SLI. One of the more common methods would be to have patients complete a binary-picture matching task, wherein the patient is presented with a sentence in both its textual and audio form and required to pair the sentence to one of two pictures which correctly portray the scenario depicted by that sentence. A standardised time delay for the presentation of the sentence, audio and binary picture exists. An audio is provided after the sentence is displayed for 5 sec and then the pictures for selection are displayed after 7 sec. Each set of questionnaires contains a fixed number of such tasks, commonly set at 40 sec. The first problem corresponding to H2-a arises because the binary picture matching task above is conducted manually. As such, the accuracy of the specific delays set in place during the diagnosis session is questionable due to potential human error. Another problem which also corresponds to H2-a is the time and cost incurred on speech language therapists who have to carry out diagnosis procedures manually. Given the problems described above, H2-a anticipates that the automation of SLI diagnosis procedures is possible and would increase diagnosis accuracy and reduce the cost of conducting said diagnosis. Proving H2-a relies significantly on conducting a thorough accuracy test which compares the time delay accuracy of an automated test to that of a manually conducted test. In our conducted experiment, the automation of an SLI diagnosis questionnaire was simulated using JavaScript (JS) structures involving the use of setTimeout (is a JS function to delay the execution of a script) for delayed presentation of a particular element.


let now = new Date();
setTimeout(() => {
  let actualDelay = (new Date()).getTime() -
  now.getTime();
  console.log(“Element   displayed:   “   +
  actualDelay);
}, elementDelay);


On the other hand, timestamps for delays in manual SLI diagnosis are recorded via JS which makes use of keydown events. For example, if a speech language pathologist thinks the delay is over and it is time to present the element on the screen, a key on the keyboard is pressed by subject and the timestamp for presenting the element is captured.


let now = new Date();
window.onkeydown = (event) => {
  let actualDelay = (new Date()).getTime() -
  now.getTime();
  console.log(“Element   displayed:   “   +
  actualDelay);
}


In this experiment, the fixed element delay was set to 5 sec — that is, elementDelay for the first script should be set to 5000 (msec). The experiment was carried out for a total of 10 times each set, for five sets. The actual time delays for both the automated diagnosis simulation and manual diagnosis simulation was recorded. The average actual time delay values for each set were compared to the perfect time delay values and the deviation percentage for each set was calculated as follows:

∣(actualDelay-elementDelay)∣/elementDelay×100%

### Singlims_ES.exe Cross-Comparison Check

In order to prove that statistical methods used to analyse SLI diagnosis results are reproducible and can be programmed into the integrated SLI diagnosis computer system, a thorough review of the SLI diagnosis process and tools utilised by speech-language pathologists has revealed that the yes-no binary classification of SLI diagnosis results are based on the comparison of an individual’s score on a single test with the score of a normative or control sample, as detailed in (7). Given the calculations and mathematical formulae involved in obtaining a proper analysis of the SLI diagnosis results, an automated yes-no binary classifier can be constructed given sufficient controlled data. The script below determines the results of attempts to implement the statistical method for single-case research delineated in (7) via JS with the jStat JS library, to obtain the one-tailed test probability value of an input test score, which plays a crucial role in determining whether the patient has SLI. Given sd = standard deviation of control sample, u = mean of control sample, N = size of control sample and score = score of test case:


function computeResult(sd, u, N, score) {
  console.log(“One-tailed probability = “ + jStat.
  ttest(((score-u)/(sd*Math.sqrt((N+1)/N))), N,
1));
}


The function above was used for random testing, and has been executed for a variety of 10 different input values for sd, u, N and score. For each execution of the script, a similar set of inputs were fed to the Singlims_ES.exe computer programme which accompanies (7).

## Results

Tables 1–3 show the results WebGazer accuracy test under the three lighting conditions, respectively, for 30 times each. To simplify the results, the average accuracy for tests conducted under the three lighting conditions were calculated and tabulated as shown in Table 4. The results of automated versus manual duration judgement test is tabulated and shown in Table 5. Singlims_ES.exe cross-comparison check is shown in Table 6.

## Discussion

The results of test 1 present some expected outcome, but also subvert quite a number of hypotheses which have been put forward prior to the accuracy test. One of the expected outcomes included gaze tracking under low lighting conditions (average accuracy: 61.379%) performing with worse accuracy as compared to global lighting (average accuracy: 88.755%). However, it was also found that WebGazer performed worst under face-focused lighting conditions (average accuracy: 52.7%). It is also worth noting that under face-focused lighting conditions, the clmtrackr component in WebGazer which is responsible for tracking face landmarks performed poorly and took a long time to recognise a face within webcam footage. This could be due to the contrasting lighting conditions between facial features and backgrounds feature due to the focused light on the face. Overall, it is safe to affirm the claim that the gaze tracking accuracy offered by WebGazer can reach 0.9 accuracy, which is within the 100px error rate. As such, it can be concluded that H1-a, given global lighting conditions, is demonstrated. By extension, the elimination of eye tracking hardware for SLI diagnosis reinforces the truthiness of H1. While it is worth noting that this study does not present any software replacement for other hardware which may be used during more advanced SLI diagnosis tests, such as EEG biosensors, the feasibility of replacing eye tracking hardware with conventional webcams doubtlessly would cut diagnosis cost and foster wider availability of SLI diagnosis.

The belief that computerised systems can be built to diagnose SLI, as introduced in H2, is far from unorthodox. In fact, many computerised tools have been developed in order to aid in data collection, visualisation and statistical calculation of diagnostical data. However, the key term of H2 is ‘integrated’, in the sense that speech language pathologists do not need multiple tools or systems to come to the conclusion of whether or not a patient has SLI but rather a single expert system to provide a definite answer given a predefined set of parameters. One of the components that can clearly be integrated is gaze locations captured via eye tracking devices during SLI diagnosis, as related to H1. In that case, machine learning techniques such as neural networks can be used to build binary classifiers to mimic expert decision making. For H2, however, the focus is placed solely on the existing difficulties faced by speech language therapists with respect to the lack of both automation and the integration of either software-related or non-computerised diagnosis tools. From the results of test 2, it is clear that automation via computers being introduced into the conventional SLI diagnosis procedures increases diagnosis accuracy in terms of the duration of judgement. For humans, the accuracy of time estimation largely depends on the length of the duration to be evaluated. For computer programmes and systems, aspects which introduce similar variables affecting timing accuracy do exist, such as CPU throttling, but the internal timers of most computer systems have a granularity of 16.666 msec (60Hz), which is a fraction of the deviation from actual time exhibited in human time estimation. We do not deny that such deviations occurring in manual SLI diagnosis procedures do not substantially affect diagnosis accuracy, but the results that we have obtained shows that there is margin for improvement, however small. From this experiment, we draw the conclusion for H2-a that the automation of the binary picture matching task for SLI diagnosis is possible, as shown in the JS script explained in the experiment details above. We also conclude that automation can introduce improvements to SLI diagnosis procedures not only in terms of cost, but also by positively impacting accuracy.

As for test 3, the one-tailed test probability values produced by both the JS script above and Singlims_ES were observed to be similar up to 3 decimal places. The script provided above can hence be safely integrated into whatever system or tool which shall be built according to the innovative approach described in this paper. We conclude that H2-b is true and by extension that H2 has been proven.

## Conclusion

Innovative techniques and approaches have frequently been proposed and prioritised within the medical world. The development of a tool or system based on the innovative approach proposed in the present study can prevent SLI from remaining the ‘hidden disorder’ by decreasing its difficulty of being diagnosed by speech-language pathologists. Based on the results of this paper, we propose that a similar integrated computer expert system can be built to help speech language pathologists diagnose and provide treatment to patients suffering from SLI.

## Figures and Tables

**Figure 1:**
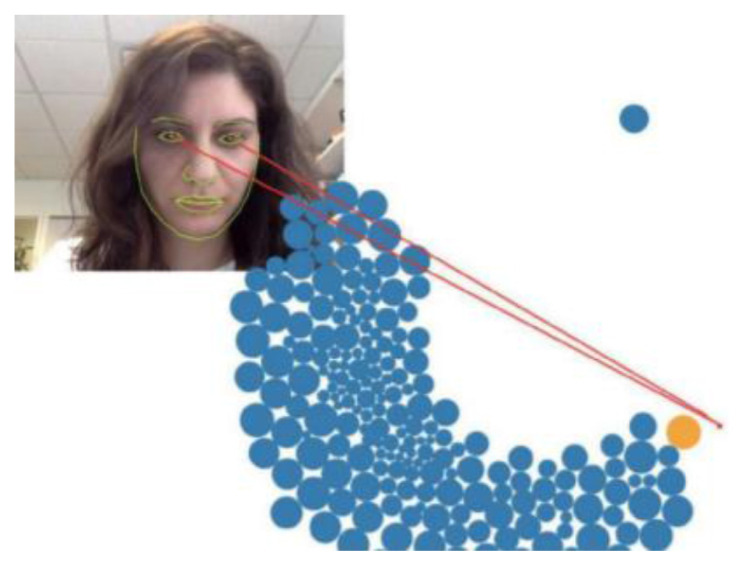
WebGazer.js in action, as seen in (5)

**Figure 2:**
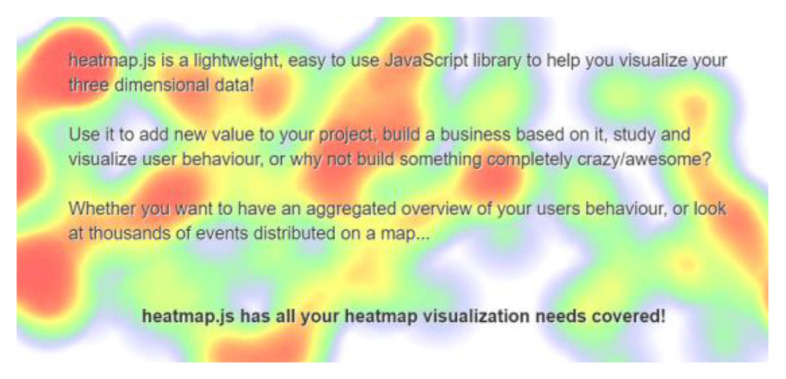
heatmap.js output, as seen in (6)

**Table 1:** WebGazer gaze tracking accuracy evaluation results (low lighting)

ID	Type	Distance (px)	Accuracy (%)
1-L	Low lighting	390.83	60.9
2-L	Low lighting	406.39	59.4
3-L	Low lighting	402.68	59.7
4-L	Low lighting	379.2	62.07
5-L	Low lighting	360.32	64
6-L	Low lighting	358.93	64.1
7-L	Low lighting	385.46	61.45
8-L	Low lighting	407.84	59.21
9-L	Low lighting	445.08	55.49
10-L	Low lighting	271.84	72.81
11-L	Low lighting	387.17	61.28
12-L	Low lighting	365.73	63.42
13-L	Low lighting	355.594	64.44
14-L	Low lighting	429.22	57.07
15-L	Low lighting	393.38	60.66
16-L	Low lighting	413.473	58.65
17-L	Low lighting	445.38	55.4
18-L	Low lighting	332.74	66.72
19-L	Low lighting	394.36	60.56
20-L	Low lighting	421.17	57.8
21-L	Low lighting	313.94	68.6
22-L	Low lighting	407.65	59.23
23-L	Low lighting	353.064	64.693
24-L	Low lighting	401.97	59.8
25-L	Low lighting	363.14	63.68
26-L	Low lighting	432.768	56.72
27-L	Low lighting	411.49	58.85
28-L	Low lighting	444.75	55.5
29-L	Low lighting	356.05	64.3
30-L	Low lighting	351.3	64.869

**Table 2:** WebGazer gaze tracking accuracy evaluation results (face-focused lighting)

ID	Type	Distance (px)	Accuracy (%)
1-FF	Face-focused lighting	456.362	54.3
2-FF	Face-focused lighting	452.91	54.7
3-FF	Face-focused lighting	454.25	54.57
4-FF	Face-focused lighting	510.69	48.93
5-FF	Face-focused lighting	372.26	62.7
6-FF	Face-focused lighting	497.01	50.29
7-FF	Face-focused lighting	579.8	42
8-FF	Face-focused lighting	515.37	48.46
9-FF	Face-focused lighting	612.29	38.7
10-FF	Face-focused lighting	443.78	55.62
11-FF	Face-focused lighting	346.58	65.34
12-FF	Face-focused lighting	489.36	51.06
13-FF	Face-focused lighting	499.36	50
14-FF	Face-focused lighting	491.8	50.8
15-FF	Face-focused lighting	482.21	51.7
16-FF	Face-focused lighting	406.06	59.3
17-FF	Face-focused lighting	517.44	48.25
18-FF	Face-focused lighting	572.14	42.7
19-FF	Face-focused lighting	457.71	54.22
20-FF	Face-focused lighting	474.94	52.5
21-FF	Face-focused lighting	475.72	52.42
22-FF	Face-focused lighting	462.08	53.7
23-FF	Face-focused lighting	452.25	54.77
24-FF	Face-focused lighting	580.539	41.94
25-FF	Face-focused lighting	403.18	59.6
26-FF	Face-focused lighting	366.18	63.3
27-FF	Face-focused lighting	492.57	50.7
28-FF	Face-focused lighting	448.32	55.1
29-FF	Face-focused lighting	310.95	68.9
30-FF	Face-focused lighting	555.79	44.42

**Table 3:** WebGazer gaze tracking accuracy evaluation results (global lighting)

ID	Type	Distance (px)	Accuracy (%)
1-G	Global lighting	96.105	90.3
2-G	Global lighting	118.58	88.14
3-G	Global lighting	103.58	89.6
4-G	Global lighting	98.95	90.1
5-G	Global lighting	120.099	87.9
6-G	Global lighting	131.469	86.85
7-G	Global lighting	117.885	88.2
8-G	Global lighting	124.2	87.5
9-G	Global lighting	67.83	93.21
10-G	Global lighting	120.06	87.9
11-G	Global lighting	95.986	90.4
12-G	Global lighting	98.726	90.12
13-G	Global lighting	90.729	90.92
14-G	Global lighting	94.73	90.5
15-G	Global lighting	121.7	87.8
16-G	Global lighting	94.79	90.52
17-G	Global lighting	125.78	87.4
18-G	Global lighting	133.56	86.6
19-G	Global lighting	100.87	89.9
20-G	Global lighting	113.76	88.62
21-G	Global lighting	123.14	87.68
22-G	Global lighting	115.93	88.4
23-G	Global lighting	139.67	86.03
24-G	Global lighting	113.67	88.63
25-G	Global lighting	114.51	88.5
26-G	Global lighting	106.96	89.303
27-G	Global lighting	99.522	90.04
28-G	Global lighting	132.40	86.7
29-G	Global lighting	112.96	88.7
30-G	Global lighting	138.17	86.18

**Table 4:** T1 average accuracy

Type	Average accuracy (%)
Low lighting	61.379
Face-focused lighting	52.7
Global lighting	88.755

**Table 5:** Accuracy test results for automated and manual diagnosis time delay

ID	Type	Average actual delay (msec)	Deviation percentage (%)
1-A	Automated	5002	0.04
2-A	Automated	5001	0.02
3-A	Automated	5001.6	0.032
4-A	Automated	5000.4	0.008
5-A	Automated	5000	0
1-M	Manual	4967	0.66
2-M	Manual	5168.5	3.37
3-M	Manual	4858.2	2.836
4-M	Manual	5041.4	0.828
5-M	Manual	5039.7	0.794

**Table 6:** Singlims_ES.exe cross-comparison check results

		Input parameters			Results (one-tailed probability)		

ID	Mean	SD	Sample size	Score	Singlims_ES	Test script	Difference
1	92.94	5.77	9	90	0.32089	0.32066	0.00023
2	93.94	5.35	8	91	0.31018	0.31022	0.00004
3	92.65	5.44	10	88	0.21805	0.21790	0.00015
4	60.91	5.12	10	80	0.00255	0.00256	0.00001
5	73.16	14.88	19	80	0.32974	0.32972	0.00002
6	76.06	11.79	33	79	0.40375	0.40376	0.00001
7	74.09	14.03	22	50	0.05396	0.05394	0.00002
8	74.75	7.41	79	70	0.26299	0.26325	0.00026
9	71.72	10.68	90	50	0.02306	0.02300	0.00006
10	74.14	8.27	81	50	0.00240	0.00240	0
